# Mental health and sexual identity inequalities in individuals with past experiences of homelessness: findings from a nationally representative survey

**DOI:** 10.1017/S2045796026100651

**Published:** 2026-04-29

**Authors:** Amal R. Khanolkar, Sally McManus, Jayati Das-Munshi, Laia Becares, Natasha Chilman

**Affiliations:** 1 Department of Population Health Sciences, Guy’s Campus, King’s College London, London, UK; 2 School of Health and Medical Sciences, City St George’s, University of London, London, UK; 3 Department of Psychological Medicine, Institute of Psychiatry, Psychology & Neuroscience, King’s College London, London, UK; 4 Population Health Improvement (PHIUK), London, UK; 5 King’s College London ESRC Centre for Society and Mental Health (CSMH), London, UK; 6 Department of Global Health and Social Medicine, Strand Campus, King’s College London, London, UK

**Keywords:** health behaviours, homelessness, mental health, self-harm, sexual minority

## Abstract

**Aims:**

Homelessness is increasing and associated with poor mental health (MH). Few studies have examined how experiences of homelessness and sexual identity intersect to effect MH. We used an intersectional approach to examine MH inequalities related to sexual identity and past homelessness in a nationally representative private household sample, and whether associations were explained by discrimination.

**Methods:**

Analysis of the 2007 and 2014 Adult Psychiatric Morbidity Surveys included 10,428 individuals aged 16–64 (58% female/3.8% non-heterosexual). The Clinical Interview Schedule-Revised (CIS-R) identified common mental disorders (CMDs). Self-harm, attempted suicide, alcohol dependence, substance use, sexual identity, discrimination/bullying, past homelessness and health behaviours were self-reported. Associations between sexual identity and homelessness were examined using multivariable Poisson regression. Prevalence ratios (PRs) for MH and health behaviours by intersectional sexual identity-past homelessness were examined using Poisson regression and adjusted for age, sex, area-level deprivation and further for discrimination/bullying.

**Results:**

Bisexual (adjusted PR [aPR]: 2.52, 95% CI: 1.48–4.29) and gay/lesbian (aPR: 1.76, 0.97–3.19) individuals were more likely to report past homelessness than heterosexual peers. Sexual minority (SM) and heterosexual individuals with past homelessness had higher prevalence of all MH outcomes compared to heterosexual peers without homelessness, with associations strongest in the SM-homelessness group (e.g., CMD: aPR: 2.67 [2.37–3.01] for heterosexual-homeless, aPR: 4.11 [3.00–5.63] for SM-homeless, aPR: 1.82 [1.45–2.28] for SM-not homeless groups), and similarly for depression/self-harm/attempted suicide. Likewise, the SM-homeless group had highest prevalence for drug dependence (aPR, 7.38 [3.15–17.29]) compared to the heterosexual-homeless (aPR, 4.03 [3.00–5.42]) and SM-not homeless (aPR, 2.19 [1.27–3.79]) groups. Adjustment for discrimination and bullying substantially attenuated point estimates, with the greatest attenuation (30–50%) in the SM-homeless compared to the heterosexual-homeless groups.

**Conclusions:**

Individuals with past experiences of homelessness have significantly worse MH than heterosexuals without homelessness, with associations highest in the SM-homeless group. Considering experiencing homelessness and SM identity together identifies a group facing particular adversity, which is often lost when examined separately. Discrimination and bullying explained much of the worse MH in SM- and heterosexual-homeless groups, but especially the former. Investigation into the mechanisms leading to MH inequalities is needed, alongside policies and services to support this group.

## Introduction

The health of people who experience homelessness constitutes an increasing global public health concern (Elder and King, 2019; Jackson *et al.*, 2024). Meta-analyses of data from high-income countries show higher rates of poor mental and physical health and co-occurring multiple conditions among populations with experiences of homelessness, contributing to premature mortality (Aldridge *et al.*, 2018; Gutwinski *et al.*, 2021; Chilman *et al.*, 2025). In England, a recent nationally representative study showed that health inequalities persist for formerly homeless people even after securing housing (Chilman *et al.*, 2024). Further research into this group has been highlighted as a priority in the UK to inform inclusion health policy and practice (Jackson *et al.*, 2024).

The higher levels of experiences of homelessness among sexual minority (SM) youth relative to heterosexual peers are well documented, with most evidence based on North American and emerging studies from Australia (Ecker, 2016; Mccarthy and Parr, 2022; Deal and Gonzales, 2023). In the UK, most evidence on higher prevalence and causes of homelessness experiences among SM youth are from convenience samples and voluntary surveys (Trust, 2015; England and Turnbull, 2024), with only one recent quantitative study which found that SM adolescents aged 17 years had higher odds to report past homelessness experiences which was associated with subsequent worse mental health (MH) compared to heterosexual peers with and without past homelessness (Khanolkar and Becares, 2025). Global studies indicate the short- and long-term impact of past homelessness experiences on mental and general health, and health behaviours like drug misuse are substantially worse among SM individuals compared to heterosexual peers who have experienced homelessness (Morton *et al.*, 2018; Ehlke *et al.*, 2022; Deal and Gonzales, 2023; Hail-Jaresa *et al.*, 2023). SM youth and young adults are more likely to experience homelessness at earlier ages, more frequently, and are more likely to engage in risky sexual behaviour to survive living with homelessness (Henny *et al.*, 2007). They are more likely to experience homelessness as a result of family rejection (due to their sexual orientation) and face added challenges like discrimination related to SM identity in homeless shelters and elsewhere and are more likely to sleep rough on the streets compared to heterosexual homeless youth (Petry *et al.*, 2022).

The evidence base on experiences of homelessness in SM adults is more limited than that on youth, but studies from the US including nationally representative data show that SM individuals are overrepresented in study samples on homelessness in adults (Ecker *et al.*, 2019; Ehlke *et al.*, 2022; Mccarthy and Parr, 2022). However, most studies are based on specific cities with significant variation in point estimates making it difficult to generalise findings (Ecker *et al.*, 2019). The first US study using national surveys found that 17.0% of SM individuals reported lifetime experiences of homelessness compared to 6.2% among heterosexual peers (Wilson *et al.*, 2020). Several factors intersect together increasing risk for homelessness in SM individuals regardless of age (Ecker *et al.*, 2020). Structural homophobia is an example of one type of stigma which leads to systemic inequalities (socioeconomic inequities, lack of legal protection and lack of data), interpersonal challenges (familial rejection, experiences of abuse) and intrapersonal challenges (such as higher rates of MH problems among SM groups in general, internalised homophobia, low self-esteem) (Ecker *et al.*, 2020). In fact, surveys conducted by UK-based charities and the government have highlighted that the main causes of homelessness experiences among SM youth include experiences of familial rejection and abuse due to SM identity (Trust, 2015; Office GE, 2024).

Substantial evidence from observational and experimental studies shows that discrimination, especially pervasive, is significantly associated with poorer MH (Emmer *et al.*, 2024). Further, the impact of pervasive discrimination is stronger in marginalised groups including SM individuals (Emmer *et al.*, 2024). Several studies show that SM individuals face different forms of discrimination (including microaggressions, stigma, differences in expectations and capabilities) across multiple environments including the workplace, residential neighbourhoods, rural areas, healthcare settings and communities with higher levels of LGBT-bias (Ayhan *et al.*, 2020; Hoy-Ellis, 2023). The impact of discrimination on MH in SM groups is also moderated by the contextual political environment (Hoy-Ellis, 2023). There now exists a substantial body of evidence showing that SM individuals with intersectional identities and experiences (like ethnic and/or gender minority, socioeconomic disadvantage, nonconformity, for example, in appearance and behaviours) face higher levels and multiple forms of discrimination associated with each identity and experience resulting in increased poorer MH compared to peers with only SM identity indicating a multiplicative relationship between discrimination and MH (Hoy-Ellis, 2023).

SM individuals are more likely to face greater challenges which impact their overall health due to experiencing discrimination, victimisation and adverse health both due to experiencing homelessness *and* being SM, i.e., substantially higher rates of worse health and adverse health behaviours due to intersectional and multiply disadvantaged identities of sexuality and being homeless (Abu-Ras *et al.*, 2021; Ehlke *et al.*, 2022). Additional and specific pathways that can lead to homelessness in SM adults are yet unclear. As most research stems from North America, it is unknown if there are specific pathways and drivers to experiencing homelessness in the SM population in the UK. Research on this in the UK (and wider Europe) is severely lacking and underdeveloped due to the lack of basic knowledge on the epidemiology of homelessness among SM adults relative to heterosexual peers. A comprehensive review by McCarthy et al. and national government-led reports have highlighted the lack of quantitative studies on experiencing homelessness and health in LGBT individuals in the UK and globally (Mccarthy and Parr, 2022; Office GE, 2024). Further, it is not known whether MH and health behaviours differ between SM adults and heterosexual peers who have experienced past homelessness. Lack of this basic knowledge hinders development of suitable public health policies to address this issue.

We hypothesise that SM individuals with past experiences of homelessness will have worse MH compared to heterosexual and SM peers with and without experiences of homelessness, respectively. Further, we also hypothesise that SM individuals with past homelessness face higher levels of discrimination (both due to past homelessness and their minoritised sexual identity) than heterosexual peers with past homelessness. Higher levels of discrimination experienced by SM individuals with past experiences of homelessness may attenuate (or partially explain) associations to a greater extent in SM individuals compared to heterosexual peers with past homelessness. The main aim of this study was to use nationally representative private household data to provide critical information on the prevalence of past experiences of homelessness by sexual orientation, and on the MH and health behaviours of people who have experienced past homelessness across sexual identity groups. As such, this study sought to (1) examine differences in self-reported past experiences of homelessness across sexual identity groups and (2) examine the MH, health behaviours and discrimination experiences of adults with intersecting experiences of SM identity and past homelessness.

## Methods

### Design and setting

The Adult Psychiatric Morbidity Survey (APMS) was initiated in 1993 and implemented roughly every 7 years to provide nationally representative data on the prevalence of treated and untreated MH problems in adults ≥16 years in England (McManus *et al.*, 2020). The repeated cross-sectional survey series – with a stratified and multi-stage random sampling design – includes individuals living in private households (non-institutional housing). Residential addresses and one adult from each address within selected primary sampling units were selected as potential participants with survey weights subsequently developed to reflect non-response and selection probabilities, to create a nationally representative sample. Participants had to speak a sufficient level of English to take part in the survey and data were collected via computer-assisted face-to-face interviews and computer-assisted self-interviewing (CASI). This analysis combines data from the 2007 and 2014 surveys and included all individuals who answered questions on sexual identity and past experiences of homelessness. Individuals ≥65 years were excluded as they were not asked about sexual identity in 2014. The eligible combined sample included 10,428 individuals (5,378 from 2007, 5,050 from 2014).

Ethical approval for the 2007 survey was provided by The Royal Free Hospital and Medical School Research Ethics Committee (reference number 06/Q0501/71). The West London National Research Ethics Committee (reference number 14/LO/0411) provided ethical approval for the 2014 survey. Data access for this analysis was approved by NHS England under a Special License from the UK Data Service (project number 263714). Data are de-identified prior to researcher access.

### Sexual identity and past experiences of homelessness

Our exposure variables were based on sexual identity and past experiences of homelessness. For analysis, we included two main exposures of interest: sexual identity (two versions) and a variable indicating intersectional experiences of sexual identity and past homelessness. Questions on sexual identity were structured slightly differently in the 2007 and 2014 surveys (Supplemental Table 1). We harmonised data across the two surveys to create a single sexual identity variable with four categories (heterosexual, bisexual, gay/lesbian and other). We created a second version of the sexual identity variable with three categories (heterosexual, SM and other), due to small sample sizes in the SM categories (bisexual and gay/lesbian).

Participants were presented with a showcard that stated eight problems or events (such as bullying, violence at work or in the home and ‘being homeless’) (Suolang *et al.*, 2021). The interviewer would then ask: ‘Now looking at this card, could you tell me if you have ever experienced any of these problems or events, at any time in your life?’, and participants were asked to identify which problem or event they experienced. Based on responses, we created a binary variable indicating none vs any past homelessness experience.

### Sexual identity and past experiences of homelessness indicator

This was created by combining the second version of the sexual identity and homelessness variables described above resulting in one variable with the following six categories: (1) Heterosexual and without past experiences of homelessness (Hetero-NH), (2) Heterosexual and with past experiences of homelessness (Hetero-H), (3) Sexual minority and without past experiences of (SM-NH), (4) Sexual minority and with past experiences of homelessness (SM-H), (5) Other and without past experiences of homelessness (Other-NH) and (6) Other and with past experiences of homelessness (Other-H). This approach allows us to estimate prevalence of outcomes in all three categories of sexual identity and homelessness experiences compared to the heterosexual group without experiences of homelessness (and consequently an *‘intercategorical’* approach in examining intersectionality between sexual identity and past homelessness and associations with later health) (Bauer *et al.*, 2021).

### Mental health

Common MH conditions, including depression and anxiety disorders, in the past week were ascertained using the structured and validated Clinical Interview Schedule-Revised (CIS-R) (Lewis *et al.*, 1992). Interviewers asked participants about common mental disorder (CMD) symptoms (i.e., non-psychotic symptoms like cognitive and emotional symptoms) over the last week, followed by more detailed questions about symptoms in the previous week. The CIS-R can be used to derive ICD-10 diagnosis of CMDs (Lewis *et al.*, 1992). A CIS-R score of ≥12 indicates presence of a CMD which would likely warrant primary care intervention. Participants’ responses to the CIS-R were also mapped to the diagnostic categories for CMDs, including depression, generalised anxiety disorder (GAD) and phobias (Lewis *et al.*, 1992; Das‐Munshi *et al.*, 2014).

Lifetime self-harm (non-suicidal) was captured by both face-to-face and CASI questions: ‘Have you ever deliberately harmed yourself but not with the intention of killing yourself?’ Responses were categorised into a binary variable (yes/no).

Lifetime attempted suicide and suicidal thoughts were also captured by both face-to-face and CASI questions: ‘Have you ever thought of taking your life, even if you would not really do it?’ and ‘Have you ever made an attempt to take your life, by taking an overdose of tablets or in some other way?’ Responses were categorised into a binary variable (yes/no).

Lifetime traumatic events or experiences (collected as part of a larger section on post-traumatic stress disorder) were ascertained by the question ‘Has a traumatic event or experience ever happened to you at any time in your life?’ followed by a brief description of possible traumatic events. Options included yes, no and do not understand/does not apply. Responses from both surveys were combined to create a binary variable indicating those without and with past traumatic experiences.

### Health behaviours

These included binary indicators of current smoking habits (yes/no), signs of drug dependence (yes/no) and frequency of any drug use (ever use; <10 times vs ≥10 times). Drinking problems were assessed using the Alcohol Use Disorder Identification Test (AUDIT) (Saunders JB, 1989), a screening tool comprising 10 questions to assess alcohol consumption, behaviours and problems (Saunders). AUDIT scores of ≥8 indicate hazardous drinking.

### Bullying and discrimination

Participants were asked about lifetime history of being bullied in The Stressful Life Events section of APMS (Brugha and Cragg, 1990). Responses were categorised into a binary variable (none/yes).

Participants were asked if they had been treated unfairly in the past year because of age, sex, ethnicity, religion, sexual orientation, physical health or MH. We created a binary indicator of any type of discrimination in the past year (none/yes). Numbers were too small to examine sub-categories of discrimination.

### Covariates

These included self-identified sex assigned at birth (male/female) and self-reported age (categorised in 10-year intervals; 16–24, 25–34, 35–44, 45–54 and 55–64 years). Due to smaller numbers of older individuals identifying as SM, we combined the 45–54- and 55–64-year age categories. Socioeconomic position (SEP) was based on the area-level Index of Multiple Deprivation (IMD). IMD is a validated and commonly used composite index of relative deprivation at small-area level known as Lower Layer Super Output Areas (LSOA) and based on seven indicators: income; employment; health deprivation and disability; education, skills and training; barriers to housing and services; crime and disorder; and living environment (Noble *et al.*, 2006). Each participant’s postcode was linked to the area’s IMD score. Total scores were categorised into quintiles (with quintile 5 being the most disadvantaged). We created a binary indicator for survey year (2007 or 2014), to capture any potential changes and differences between the two sweeps.

Component items of each scale, all MH and health behaviours, and how they were operationalised for analysis are listed in Supplemental Table 1.

### Statistical analysis

We first estimated differences in proportions of all outcomes across the six categories of the sexual identity and homelessness indicator variable.

Differences in individuals with past homelessness experiences by sexual identity were estimated using Poisson regression models with a log-link function; Model 1 was run unadjusted, followed by Model 2 adjusted for age, sex, SEP and sweep.

The second set of regression models examining differences in MH, health behaviours, bullying and discrimination used the sexual identity and past experiences of homelessness indicator as the exposure variable. We first estimated prevalence ratios (PRs) for all outcomes for each of the five sexual identity and past homelessness status categories compared to the reference heterosexual and without past experiences of homelessness category using Poisson regression models with a log-link function. PRs were first estimated unadjusted (Model 1) and secondly adjusted for age, sex, ethnicity, SEP (IMD) and survey year (Model 2). We additionally adjusted for discrimination and bullying (Model 3) for MH and health behaviours only (to assess whether inequalities in MH/health behaviours are explained by past discrimination and bullying).

All analyses were run on complete case samples. There were no missing data on age, sex and IMD. Final sample sizes varied depending on missing data on outcome variable (<1% of the eligible sample). All analyses were conducted using Stata version 18. All models included survey weights which accounted for the complex survey design and non-response, ensuring estimates for associations were representative of the household population in England. Models were run with the ‘svy’ package in Stata to account for the survey design and ensure robust estimates of variance.

## Results

A total of 538 (5.0%) individuals reported past experiences of homelessness. Bisexual (13.9%), gay/lesbian (7.9%) and other (7.5%) adults were more likely to report past experiences of homelessness compared to heterosexual peers (4.8%, *p* < 0.001), Table 1. Past homelessness experiences were also more common among socioeconomically deprived groups with a gradient of increasing past homelessness with increasing deprivation (2.1% in IMD quintile 1 vs 8.6% in quintile 5, *p* < 0.001). There were no differences in past homelessness experiences by sex or sweep year (Table 1).

**Table 1.** Distribution of sexual identity and covariates by past experiences of homelessness in 10,675 individuals who answered the 2007 or 2014 Adult Psychiatric Morbidity Surveys (APMS)

	Past homelessness		
	No	Yes	
	*N* = 10,137 (95.9%)	*N* = 538 (5.1%)	*p*-value^a^
	**%**	**%**	
**Sexual identity**			
Heterosexual	95.22	4.78	*p* < 0.001
Bisexual	86.09	13.91	
Gay/lesbian	92.02	7.98	
Other	92.47	7.53	
**Sex**			
Male	94.67	5.33	*p* = 0.250
Female	95.17	4.83	
**Socioeconomic deprivation**			
Quintile 1	97.94	2.06	*p* < 0.001
Quintile 2	96.83	3.17	
Quintile 3	95.23	4.77	
Quintile 4	93.66	6.34	
Quintile 5 (*most disadvantaged*)	91.39	8.61	
**Age (years)**			
16–24	95.63	4.37	*p* < 0.005
25–34	93.59	6.41	
35–44	94.70	5.3	
45–64	95.52	4.48	
**Sweep**			
2007	95.19	4.81	*p* = 0.282
2014	94.73	5.27	

a *p*-value for Chi^2^ test for differences in proportions across categorical variables.

Table 2 displays the prevalence of MH outcomes and health behaviours by sexual identity and past homelessness experiences. For most MH outcomes, individuals with past experiences of homelessness had higher proportions of adverse MH compared to peers without past homelessness. However, the group with SM identity *and* past experiences of homelessness consistently had the highest proportions with adverse MH and mostly higher than the SM group without past-homelessness. For example, the prevalence of CMD varied from 18.1% and 49.2% in heterosexuals without and with past-homelessness, respectively, to 30.1% and 72.4% in SM groups without and with past-homelessness, respectively. Similarly, prevalence of self-harm was lowest in heterosexuals without past-homelessness (5.2%) increasing to 21.7% in SM individuals without past-homelessness, 26.8% in heterosexuals with past-homelessness and highest in SM individuals with past-homelessness (34.5%) group.

**Table 2.** Prevalence estimates of health conditions, discrimination, bullying and health behaviours for individuals who took part in the 2007 or 2014 Adult Psychiatric Morbidity Survey (APMS), by past experiences of homelessness and sexual identity

		Heterosexual & no homelessness	Heterosexual & past homelessness	Sexual minority & no homelessness	Sexual minority & past homelessness	Other & no homelessness	Other & past homelessness	Total	*p*-value^a^
**CMD**	No	7806	243	174	^b^<10	104	^b^<10	8339	<0.005
		*81.94*	*50.84*	*69.88*	*27.59*	*77.04*	*36.36*	*79.97*	
	Yes	1720	235	75	21	31	^b^<10	2089	
		*18.06*	*49.16*	*30.12*	*72.41*	*22.96*	*63.64*	*20.03*	
**GAD**	No	8997	388	225	22	124	10	9766	<0.005
		*94.45*	*81.17*	*90.36*	*75.86*	*91.85*	*90.91*	*93.65*	
	Yes	529	90	24	7	11	^b^<10	662	
		*5.55*	*18.83*	*9.64*	*24.14*	*8.15*	*9.09*	*6.35*	
**Depressive episode**	No	9202	404	230	20	126	^b^<10	9991	<0.005
		*96.6*	*84.52*	*92.37*	*68.97*	*93.33*	*81.82*	*95.81*	
	Yes	324	74	19	^b^<10	10	^b^<10	437	
		*3.4*	*15.48*	*7.63*	*31.03*	*6.67*	*18.18*	*4.19*	
**Phobia**	No	9292	415	233	24	131	10	10,105	<0.005
		*97.54*	*86.82*	*93.57*	*82.76*	*97.04*	*90.91*	*96.9*	
	Yes	234	63	16	^b^<10	^b^<10	^b^<10	323	
		*2.46*	*13.18*	*6.43*	*17.24*	*2.96*	*9.09*	*3.1*	
**Self-harm**	No	9029	350	195	19	123	^b^<10	9725	<0.005
		*94.8*	*73.22*	*78.31*	*65.52*	*91.11*	*81.82*	*93.28*	
	Yes	495	128	54	10	12	^b^<10	701	
		*5.2*	*26.78*	*21.69*	*34.48*	*8.89*	*18.18*	*6.72*	
**Attempted suicide (lifetime)**	No	7725	199	146	^b^<10	105	^b^<10	8185	<0.005
		*81.14*	*41.81*	*58.63*	*13.79*	*77.78*	*54.55*	*78.55*	
	Yes	1,795	277	103	25	30	^b^<10	2235	
		*18.86*	*58.19*	*41.37*	*86.21*	*22.22*	*45.45*	*21.45*	
**Past traumatic experiences**	No	5856	171	121	^b^<10	90	^b^<10	6247	<0.005
		*62.85*	*36.23*	*50.21*	*14.29*	*72*	*50*	*61.29*	
	Yes	3461	301	120	24	35	^b^<10	3946	
		*37.15*	*63.77*	*49.79*	*85.71*	*28*	*50*	*38.71*	
**Discrimination**	No	8755	352	181	17	106	10	9420	<0.005
		*92.09*	*74.11*	*72.69*	*58.62*	*80.3*	*82*	*90.55*	
	Yes	752	123	68	12	26	^b^<10	983	
		*7.91*	*25.89*	*27.31*	*41.38*	*19.7*	*18*	*9.45*	
**Bullying**	No	7209	219	130	10	106	^b^<10	7681	<0.005
		*75.68*	*45.82*	*52.21*	*34.48*	*78.52*	*63.64*	*73.66*	
	Yes	2317	259	119	19	29	^b^<10	2747	
		*24.32*	*54.18*	*47.79*	*65.52*	*21.48*	*36.36*	*26.34*	
***Health behaviours***									
**Current smoking**	No	7339	234	168	10	103	^b^<10	7860	<0.005
		*77.04*	*48.95*	*67.47*	*34.48*	*76.3*	*54.55*	*75.37*	
	Yes	2187	244	81	19	32	^b^<10	2568	
		*22.96*	*51.05*	*32.53*	*65.52*	*23.7*	*45.45*	*24.63*	
**Problem drinking**	No	7445	346	176	17	111	^b^<10	8103	<0.005
		*78.25*	*72.38*	*70.68*	*58.62*	*82.22*	*72.73*	*77.79*	
	Yes	2069	132	73	12	24	^b^<10	2313	
		*21.75*	*27.62*	*29.32*	*41.38*	*17.78*	*27.27*	*22.21*	
**Drug dependence**	No	9160	386	227	22	127	10	9932	<0.005
		*97.41*	*84.65*	*91.9*	*78.57*	*94.07*	*90.91*	*96.61*	
	Yes	244	70	20	^b^<10	11	^b^<10	349	
		*2.59*	*15.35*	*8.1*	*21.43*	*5.93*	*9.09*	*3.39*	
**Drug frequency**	0–9	8858	348	191	19	127	^b^<10	9552	<0.005
		*93.02*	*72.96*	*76.71*	*65.52*	*94.07*	*81.82*	*91.63*	
	≥10	665	129	58	10	10	^b^<10	872	
		*6.98*	*27.04*	*23.29*	*34.48*	*5.93*	*18.18*	*8.37*	

a p-value for Chi^2^ or Fisher’s exact test for differences in proportions across categorical variables. CMD: common mental disorder, GAD: generalised anxiety disorder.

b Suppressed as N<10.

A similar pattern was observed for all health behaviours (for example, the prevalence of current smoking varied from 22.9% and 51.1% in heterosexuals without and with past-homelessness, respectively, to 32.5% and 65.5% in SM groups without and with past-homelessness, respectively).

Prevalence of past discrimination and bullying varied by both sexual identity and past experiences of homelessness, and was always highest amongst SM individuals. Individuals with past experiences of homelessness reported higher levels of discrimination; 41.4% in SM group with past-homelessness and 25.9% in the heterosexual group with past-homelessness compared to 7.9% in the heterosexual group without homelessness. Similarly, bullying was highest in the SM (65.5%) and heterosexual (54.2%) groups with past-homelessness compared to heterosexual (24.3%) and SM (47.8%) peers with past-homelessness.

**Figure 1.**
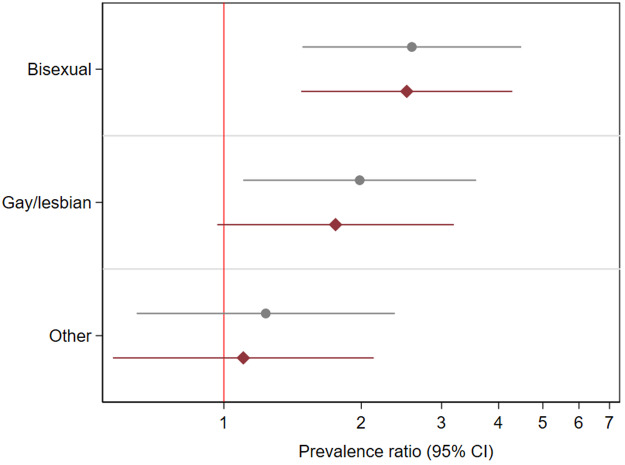
Associations between sexual identity and past experiences of homelessness in 10,428 individuals who answered the 2007 or 2014 Adult Psychiatric Morbidity Surveys (APMS).

*Notes:* Reference category: Heterosexual individuals. Estimates are from multivariable Poisson regression models.

SM individuals (bisexual: PR: 2.58, 95% CI: 1.49–4.48 and gay/lesbian: PR: 1.98, 1.10–3.57, 1.09–3.94, Supplemental Table 2 and Fig. 1) had higher prevalence of past homelessness experiences than heterosexual peers. Adjustment for covariates marginally attenuated associations (and no longer evident for gay/lesbian individuals).

**Figure 2.**
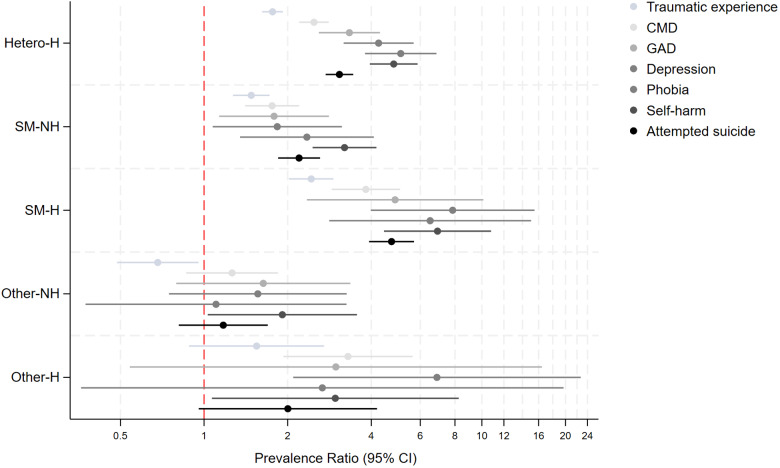
Prevalence of mental health problems based on past experiences of homelessness and sexual identity in 10,428 individuals who answered the 2007 or 2014 Adult Psychiatric Morbidity Surveys.

*Notes:* Hetero-H: Heterosexual and past homelessness; SM-NH: Sexual minority and no past homelessness; SM-H: Sexual minority and past homelessness; Other-NH: Other sexual identity and no past homelessness; Other-H: Other sexual identity and past homelessness; Reference category: Heterosexual individuals and no past homelessness; Estimates are from multivariable Poisson regression models.

Supplemental Table 3 and Figure 2 display the crude and adjusted PRs (aPRs) from Poisson regression models examining associations between the sexual identity and past homelessness experiences indicator and MH outcomes. Adjustment for age, sex, survey year and SEP (i.e., Model 2) only marginally attenuated associations. Compared to heterosexuals without past-homelessness, individuals with past experiences of homelessness had two to eight times higher prevalence of all MH outcomes, irrespective of sexual identity. SM groups with and without past experiences of homelessness and the heterosexual group with past-homelessness had consistently higher aPR for all MH outcomes, with higher point estimates observed for both the heterosexual and SM groups with past homelessness. However, the highest point estimates were observed for the SM group with past homelessness for all outcomes. For example, the SM group with past homelessness had four to eight times significantly higher prevalence of CMD (aPR: 3.81, 95% CI: 2.86–5.08), depression (aPR: 7.77, 3.89–15.50), lifetime traumatic experiences (aPR: 2.30, 1.91–2.79), self-harm (aPR: 7.81, 4.90–12.43) and attempted suicide (aPR: 4.96, 3.98–6.17) compared to the heterosexual group without past-homelessness and significantly higher than the heterosexual with past-homelessness and SM without past-homelessness groups. Findings for the other-SM group were less consistent.

Adjustment for discrimination and bullying (Model 3 in Supplemental Table 3, Supplemental Figure 1) attenuated the sexual identity-past homelessness and MH associations by 20–60%, with the greatest attenuation observed in the SM with past-homelessness group. For example, for CMD, adjustment for discrimination/bullying attenuated point estimates from aPR 2.49–1.82 in heterosexual with past-homelessness and aPR 3.82–2.42 in SM with past-homelessness groups. For most MH outcomes, associations remained significant after adjustment for discrimination and bullying but point estimates were attenuated by 30–50% in the SM with past-homelessness groups for GAD, depression, phobias, self-harm and attempted suicide.

**Figure 3.**
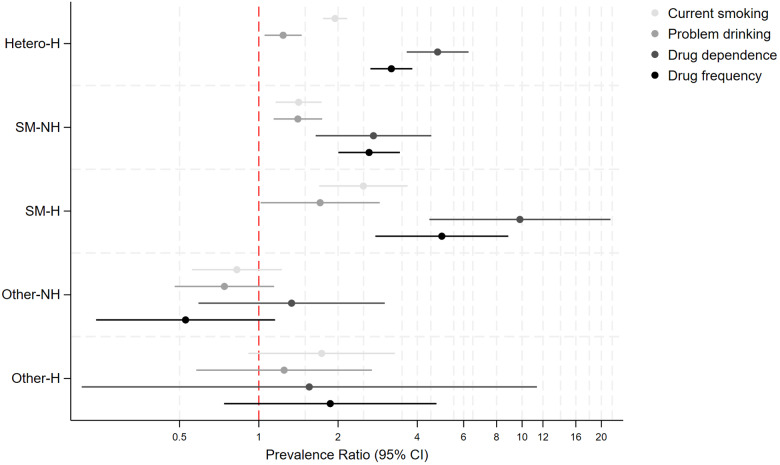
Prevalence of health behaviours based on past experiences of homelessness and sexual identity in 10,428 individuals who answered the 2007 or 2014 Adult Psychiatric Morbidity Surveys.

*Notes:* Hetero-H: Heterosexual and past homelessness; SM-NH: Sexual minority and no past homelessness; SM-H: Sexual minority and past homelessness; Other-NH: Other sexual identity and no past homelessness; Other-H: Other sexual identity and past homelessness; Reference category: Heterosexual individuals and no past homelessness; Estimates are from multivariable Poisson regression models.

Supplemental Table 4 and Figure 3 display crude and aPR for health behaviours. A similar pattern was observed for health behaviours, with both heterosexual and SM with past-homelessness individuals having consistently higher aPR for all health behaviours compared to heterosexual individuals without past-homelessness, with the highest aPR for the SM with past-homelessness group (e.g., drug dependence: Hetero-H: aPR: 3.14, 2.62–3.77, SM-NH: aPR: 2.59, 1.98–3.39, SM-H: aPR: 4.84, 2.68–8.73). Prevalence was four to nine times higher for drug dependence and higher drug frequency in the SM with past-homelessness group, compared to the heterosexual individuals without past-homelessness group. Adjustment for covariates marginally attenuated point estimates, and adjustment for discrimination and bullying did not substantially attenuate point estimates as observed for MH outcomes. There were no significant differences in prevalence of health behaviours for the other sexual identity group.

Table 3 shows crude and aPR for bullying and discrimination. Individuals with past experiences of homelessness had 3–4.5 times higher prevalence (heterosexual with past-homelessness; aPR: 2.82, 2.30–3.46, SM with past-homelessness; aPR: 4.69, 2.86–7.69) for experiencing discrimination compared to heterosexual individuals without past-homelessness. The SM group without past homelessness, SM and heterosexual groups with past homelessness had significantly higher aPR for experiencing past bullying, but differences in the point estimates’ sizes were minimal.

**Table 3.** Differences in experiences of discrimination and bullying by sexual identity and past homelessness in 10,428 individuals who took part in the 2007 or 2014 Adult Psychiatric Morbidity Survey (APMS)

	Discrimination				Bullying			
	Model 1		Model 2		Model 1		Model 2	
	PR	95% CI	PR	95% CI	PR	95% CI	PR	95% CI
**Hetero-NH**	**1**		**1**		**1**		**1**	
**Hetero-H**	**2.83**	**2.31, 3.47**	**2.82**	**2.30, 3.46**	**2.16**	**1.94, 2.41**	**2.19**	**1.96, 2.45**
**SM-NH**	**3.42**	**2.67, 4.37**	**3.07**	**2.40, 3.92**	**2.02**	**1.73, 2.37**	**1.92**	**1.63, 2.25**
**SM-H**	**4.28**	**2.57, 7.15**	**4.69**	**2.86, 7.69**	**2.53**	**1.77, 3.62**	**2.49**	**1.78, 3.48**
**Other-NH**	**2.89**	**1.97, 4.25**	**2.74**	**1.87, 4.00**	0.91	0.63, 1.34	0.92	0.63, 1.35
**Other-H**	2.22	0.57, 8.62	1.56	0.42, 5.74	1.56	0.68, 3.56	1.53	0.71, 3.29
**Observations**	10,403		10,403		10,428		10,428	

*Note:* NH: no experiences of past homelessness, H: past experiences of homelessness, SM: sexual minority. Model 1: Unadjusted Poisson regression. Model 2: Poisson regression adjusted for age, sex, socioeconomic deprivation (IMD) and sweep (2007 or 2014). Text in bold text indicates 95% confidence intervals that do not include 0.

## Discussion

We found that SM individuals with past experiences of homelessness had significantly higher prevalence of a range of MH conditions and adverse health behaviours compared to heterosexual peers without and with experiences of past homelessness. *A consistent pattern was found with SM individuals with past-homelessness having the highest prevalence for most MH conditions* (four- to fivefold higher prevalence for CMDs, GAD and lifetime attempted suicide and seven- to eightfold higher prevalence for depression and self-harm), compared to heterosexual peers without past homelessness experiences. *A pertinent finding was that adjustment for past discrimination and bullying significantly attenuated associations by 20–60% for all MH outcomes with the greatest attenuation observed for the SM-H group.* A similar pattern was observed for drug dependence and greater frequency of drug use. In general, we found a consistent gradient of increasing prevalence for most MH outcomes and some health behaviours increasing in effect size from SM without past-homelessness to heterosexual and SM groups with past-homelessness. We observed a similar gradient for experiences of past discrimination (>4 fold and >2.5 fold higher in SM and heterosexual groups with past-homelessness, respectively) by sexual identity and past homelessness. In line with our hypothesis, our findings demonstrate substantial inequalities in MH, health behaviours, and experiences of discrimination and bullying at the intersection of sexual identity and past experiences of homelessness, which would otherwise remain hidden if examined separately. Further, both SM and heterosexual individuals with past homelessness experience high levels of discrimination which explain a substantial portion of the poor MH in the SM with past-homelessness group.

### Comparison with literature

This is the first study in the UK to quantitatively examine risk for past experiences of homelessness and MH in SM adults using an intersectional approach in a nationally representative sample. As such this limits comparison with UK- and European-based studies. Internationally, the evidence base on homelessness and MH in SM adults is limited being largely restricted to younger groups (<25 years of age) with most studies focusing on North American data. In the UK, due to longstanding under-investment in data collection that allows for adequately sampled populations of both SM and homeless groups, the existing evidence base largely consists of studies using convenience samples, restricted to younger ages and selected geographical locations. The first comprehensive UK-based LGBTQ+ Housing and Homelessness Survey was conducted in 2022–24 and included >1100 LGBTQ+ adults (England and Turnbull, 2024). The survey found that 47% of participants had experienced lifetime homelessness and 20% had experienced homelessness in the past year. While this survey provided critical information on homeless experiences including reasons and differences by key demographic factors like age and ethnicity, it did not include a heterosexual comparator group and data on MH and health behaviours. The only other data on prevalence of homelessness experiences by sexual identity are from the 2021 national census which found that 7.7% of SM adults had experienced homelessness, more than double the proportion of 3.2% of individuals who identified as SM in the census (ONS, 2023a, 2023b).

### Mechanisms and explanations

The intersectionality framework theory predicates that individuals with ≥2 minority identities experience unique forms of stigma and discrimination which originate at the intersections of minoritised identities situated within broader societal systems of power and oppression (Kimberly, 1989). Thus, SM individuals who are homeless encounter a unique set of problems (like stigma and discrimination) associated with both or at the intersection of these identities and/or experiences as compared to heterosexual and SM peers with and without homelessness experiences. These intersectional experiences can have greater impact on subsequent MH, well-being and health behaviours in marginalised individuals including SM groups (DeSon and Andover, 2024). While the evidence base on past homelessness and subsequent MH and health behaviours in SM adults is still emerging, limited but crucial qualitative studies have highlighted unique experiences encountered by SM individuals who have experienced homelessness (Adley *et al.*, 2025). As highlighted in a recent systematic review of UK-based qualitative studies, examples include ‘normative practises’ (SM groups not being included in the ‘normal remit’ of housing services and thus being shuttled between different services), ‘lack of cultural awareness’ (services not wanting to address SM identities or not understanding potential implications and considerations of being SM) and SM individuals ‘anticipating’ negative experiences with accessing formalised services and help due to fear of being discriminated against by both service providers and other users, and services generally being designed or orientated for normative populations (Adley *et al.*, 2025). This was often due to hearing about experiences from peers when they had accessed homeless services and thus many SM individuals seek alternatives like sleeping rough or temporary solutions like ‘couch surfing’ or moving frequently between friends who provide help (Adley *et al.*, 2025). Even when services, governmental and alternative, provide help and/or are LGBTQ+ friendly, discrimination faced from staff and/or other homeless individuals is common (McCann and Brown, 2019; Ecker *et al.*, 2020; Mccarthy and Parr, 2022). Further, the number of services designed for LGBTQ+ individuals are often limited in number and restricted to larger urban cities (Mccarthy and Parr, 2022). Thus, SM individuals face unique barriers and challenges when they experience homelessness and the ramifications of this on subsequent MH are potentially different from heterosexual peers. Our findings illustrate severe MH inequalities for people with past experiences of homelessness who are gay, lesbian and/or bi-sexual and in line with the intersectionality framework – i.e., SM individuals with these intersecting experiences face the widest inequalities with respect to the outcomes studied.

The present study also draws attention to experiences of discrimination for the SM group. This is unsurprising as experiences of discrimination have previously been reported by young SM people with experience of homelessness (Shelton *et al.*, 2018; McCann and Brown, 2019; Ehlke *et al.*, 2022). However, the present study extends this finding to adults in England who have previous experience of homelessness. Homophobia and heterosexism may play an important structural role in impacting entries into and exits from homelessness for people in SM groups (Ecker *et al.*, 2019) and could, therefore, be a target for preventative policies to reduce inequalities.

We acknowledge that the data used in this study were collected over 10 years ago at time of writing. Since this time, many countries including the UK have experienced significant and positive changes in societal attitudes encompassing greater acceptance and understanding of sexuality and positive legislation (e.g., The Equality Act 2010 and The Marriage Act 2013 in the UK) for SM rights. However, individuals with homelessness experiences continue to report worse MH (Vickery *et al.*, 2021). There is robust evidence that despite societal changes and national laws aiming to prevent identity and experience related discrimination, minoritised groups continue to report worse MH (Khanolkar, 2025; Khanolkar *et al.*, 2023). However, the lack of contemporary data to study the intersection of homelessness and sexual identity is a substantial barrier and currently the APMS is the only nationally representative data in England available to study our research aims. We believe that our findings are relevant to the present day as both SM and homeless groups continue to face substantial discrimination and barriers.

### Strengths and limitations

This study has strengths including a large nationally representative sample with low missing data allowing generalisability to adults living in private households in England. CMDs and possible alcohol dependence were assessed using validated and widely used instruments which enable comparisons with other studies that have used the same instruments. Sexual identity was self-reported and sensitive information like health behaviours was collected by CASI which could make participants feel less stigmatised in reporting such information. We used PRs which are more reliable and easier to understand compared to odds ratios, which can overestimate effect sizes especially when the outcome is common like with MH conditions and common health behaviours (Thompson *et al.*, 1998). Our methodological approach of combining sexual identity and past homelessness exposure enabled us to incorporate testing for interactions and consequently the *‘intercategorical’intersectional approach’* (and more specifically apply the intersectionality framework) in examining whether MH and health behaviours differed between sexual identity and past homelessness groups (Bauer *et al.*, 2021).

There are limitations that need to be acknowledged. The cross-sectional design precludes conclusions on causality. Data on both homelessness and all outcomes were collected at the same time limiting temporality. Nonetheless, we hypothesised that for most individuals, past homelessness predates the CMD studied here. The smaller number (3.8%) of non-heterosexual participants resulted in relatively small numbers of SM individuals with past homelessness impacting the precision (i.e., wider confidence intervals) of some point estimates. However, despite the wide confidence intervals for some outcomes, we observed a general pattern of worse MH among SM individuals with past homelessness compared to SM individuals without and heterosexual peers with past homelessness. The wider confidence intervals observed for the SM groups are most likely due to low statistical power, but we advise caution in interpretation. Further, the proportion of individuals identifying as SM is similar to that reported in 2021 national census (2023). We were unable to examine duration, number of times of and reason for homelessness which can differ between SM and heterosexual individuals. Further, we could not examine differences (including testing for interaction or moderation) by ethnicity, sex/gender and age due to small numbers. Several studies have showed substantial variation in risk for worse MH and higher levels of health behaviours in SM subgroups. For example, bisexual individuals consistently report worse MH compared to gay and lesbian peers. Bisexual and female SM groups report higher levels of some health behaviours like smoking and substance use compared to male SM groups. We were unable to examine differences by SM subgroup due to small numbers which necessitated combining all SM groups together for analysis on MH and health behaviour outcomes (Marshal *et al.*, 2008; Wittgens *et al.*, 2022). These need to be examined in future studies where data allow it. All outcomes were based on self-reported data and there is a possibility that participants may not accurately report information (for example, social desirability bias or not want to recall particularly distressing events like self-harm) which could lead to underestimation of prevalences. Some individuals may not want to think about past distressing events like self-harm, attempted suicide and traumatic events which could result in underestimation, but we do not think this will significantly vary by sexual identity. We found evidence for significantly worse MH (like CMD and depression) and discrimination in the ‘other’ sexual identity group, and in general the size and direction of effect estimates suggested they had higher risk for all MH outcomes though not statistically significant due to small numbers making it difficult to make firm conclusions. A previous UK study showed that the ‘other’ sexual identity group is heterogenous and likely includes individuals who are unsure of their sexual identity or identify with other SM groups like asexual/pansexual/demisexual etc. (Khanolkar, 2025). Nonetheless, it is vital that future national surveys include more options for sexual identities and oversample SM groups in general.

Data used in this study date from 2007 and 2014, and rates of homelessness have increased since then and further exacerbated by the COVID-19 pandemic and the housing crisis in England. Proportions of the population identifying as SM have also increased. Shelter, a national charity working with homeless individuals found that homelessness increased by 14% between 2023 and 2024 (SHELTER, 2024). We were unable to include individuals over 65 years as they were not asked about sexual identity. Despite the CISR-R and GAD instruments being developed a while back, they remain clinically relevant and used in both clinical and research settings and also enable studying population change over time as in this study.

Individuals who had experiences of homelessness in the period when the survey was conducted are not included in our sample, as by design the APMS included individuals living in private households. Thus, individuals for example living in homeless shelters or rough sleeping would be excluded, and this sample does not represent those who are currently experiencing or who have had long-term experiences of homelessness, who are likely to have worse health outcomes. Lastly, we were unable to examine SM-specific risk factors that can lead to homelessness and worse MH in this group due to lack of data.

Sexual orientation can be conceptualised and ascertained in multiple ways including self-identification (as used in the APMS and other common surveys and studies in the UK), behaviour and attraction, or a combination of these (Sell, 1997). Further, an individual’s sexual orientation may also change across the life course. In this study, we used self-identified sexual identity which may not capture all individuals with same-sex sexual behaviour or those who may no longer identify as SM for example. Future studies should examine sexual orientation related differences in homelessness and subsequent MH using broader definitions than that of only sexual identity where data permit.

To conclude, this study found pronounced MH inequalities and discrimination experienced by heterosexual and SM individuals with past experiences of homelessness, compared to heterosexual peers without past experiences of homelessness. Further, we observed a strong pattern suggesting that SM individuals with past experiences of homelessness had worse MH and higher levels of discrimination compared to SM and heterosexual peers without and with past experiences of homelessness, respectively. A similar pattern was observed for health behaviours. Importantly, discrimination and bullying explained a substantial portion of the increased MH inequalities experienced by individuals with past homelessness, and especially in the SM group. There is an urgent need to develop policies, interventions and clinical services to provide tailored support for individuals with past experiences of homelessness which can reduce and prevent inequalities. Our findings indicate that policies should also account for sexual identity as SM individuals face unique identity related barriers and discrimination not experienced by heterosexual peers. Experiencing discrimination is a significant risk factor for poor MH, and policies that tackle discrimination need to be strengthened. However, significantly more needs to be done in collecting more relevant and contemporary data with adequate sample sizes which will enable studying relationships and pathways between SM status, homelessness and MH.

## Supporting information

10.1017/S2045796026100651.sm001 Khanolkar et al. supplementary material Khanolkar et al. supplementary material

## Data Availability

Data for this study are from the 2007 and 2014 Adult Psychiatric Morbidity Surveys (APMS). APMS (deidentified data) data are available to use for researchers (free of cost) and can be accessed from the UK Data Service website: https://ukdataservice.ac.uk/. Relevant data dictionaries and survey reports for each APMS survey can be found on the UK Data Service website. Prospective users need to register on the UK Data Service website and agree to the necessary licence terms and conditions. For accessing the APMS data, prospective users must fill out the Special Licence User Agreement which is verified by the data custodians before access to the data is granted. Syntax/code used for the analysis in this study is available on request from the corresponding author.

## Supplementary material

The supplementary material for this article can be found at https://doi.org/10.1017/S2045796026100651.
