# Supplementary material for: Mental health and sexual identity inequalities in individuals with past experiences of homelessness: findings from a nationally representative survey
Source: Epidemiol Psychiatr Sci. 2026 Apr 29;35:e28. doi: 10.1017/S2045796026100651 (PMC13126408; doi:10.1017/S2045796026100651)
Supplement: Khanolkar et al. supplementary material [file S2045796026100651sup001.docx]

**Supplementary Data for Mental health and sexual identity inequalities in individuals with past experiences of homelessness: Findings from a nationally representative survey**

**Contents:**

**Supplemental Table 1………………………………………………….2**

**Supplemental Table 2………………………………………………….5**

**Supplemental Table 3………………………………………………….6**

**Supplemental Table 4………………………………………………….8**

**Supplemental Figure 1………………………………………………10**

**Supplemental Table 1.** **A detailed description of all variables including exposures, mental health and health behaviours assessed in the 2014 Adult Psychiatric Morbidity Survey (APMS)**

| **Variable** | **Original question in 2014 survey** | **Final form used in analysis** | **Notes** |
| --- | --- | --- | --- |
| **Homelessness** | SHOW CARD N5  *Now looking at this card, could you tell me if you have ever experienced any of these problems or events, at*  *any time in your life:*  1 Bullying  2 Violence at work  3 Violence in the home  4 Sexual abuse  5 Being expelled from school  6 Running away from your home  7 **Being homeless**  8 None of these | *Binary:*  No vs yes | Those who chose option 7 (being homeless) were considered to have had past experiences of homelessness |
| **Sexual identity** | *Which of the following options best describes how you think of yourself?*  1 Heterosexual or Straight  2 Gay or Lesbian  3 Bisexual  4 Other | *Categorical:*  Heterosexual  Gay/lesbian  Bisexual  Other  2^nd^ version:  Heterosexual  Sexual minority  Other | First version used in analysis on sexual identity and past homelessness  Second version used in analysis on mental health & health behaviours |
| **Common mental disorders (CMD)** | Any CMD present, indicated by a score ≥12 on the Clinical Interview-Schedule-Revised scale, which asks after symptoms in the last month and week. | Binary:  None vs yes |  |
| **Generalised anxiety disorder (GAD)** | Individuals meeting criteria for: 1. Duration ≥6 months 2. Free-floating anxiety 3. Autonomic over-activity 4. Overall anxiety score ≥2. | Binary:  None vs yes |  |
| **Depression** | Any depressive episode present | Binary:  None vs yes |  |
| **Past traumatic experiences** | *Has a traumatic event or experience ever happened to you at any time in your life?*  *The term traumatic event or experience means something like a major natural disaster, a serious automobile*  *accident, being raped, seeing someone killed or seriously injured, having a loved one die by murder or suicide,*  *or any other experience that either put you or someone close to you at risk of serious harm or death.*  *Has a traumatic event or experience ever happened to you at any time in your life?*  1 Yes  2 No  9 Don’t Understand/Does Not Apply | Binary:  None vs yes |  |
| **Suicide (lifetime)** | *There may be times in everyone's life when they become very miserable and depressed and may feel like*  *taking drastic action because of these feelings.*  *Have you ever thought of taking your life, even if you would not really do it?*  1 Yes  2 No  *Have you ever made an attempt to take your life, by taking an overdose of tablets or in some other way?*  1 Yes  2 No | Binary:  No vs yes |  |
| **Self-harm** | *Have you ever deliberately harmed yourself in any way but not with the intention of killing yourself?*  1 Yes  2 No  Did you…  You may give more than one response  1 Cut yourself  2 Or burn yourself  3 Or swallow anything  4 Or harm yourself some other way  IF DSHharm = Yes  DSH7  Did you do any of these things to draw attention to your situation or to change your situation?  1 Yes  2 No  IF DSHharm = Yes  DSH8  Did you do any of these things because it relieved unpleasant feelings of anger, tension, anxiety or  depression?  1 Yes  2 No  IF DSHharm = Yes  DSH9  Have you received medical attention for deliberately harming yourself in any of these ways?  1. Yes  2. No  IF DSHharm = Yes  DSH10  Have you ever seen a psychiatrist, psychologist or counsellor because you had harmed yourself?  1 Yes  2 No | Binary:  No vs yes |  |
| **Discrimination** | *The next questions are about whether you have been unfairly treated in any aspect of your life, because you*  *belong to a particular group.*  *Have you been unfairly treated in the last 12 months*  *because of your skin colour or ethnicity?*  1 Yes  2 No  *Have you been unfairly treated in the last 12 months*  *because of your sex?*  1 Yes  2 No  *Have you been unfairly treated in the last 12 months*  *because of your religious beliefs?*  1 Yes  2 No  *Have you been unfairly treated in the last 12 months*  *because of your age?*  1 Yes  2 No  *Have you been unfairly treated in the last 12 months*  *because of your mental health?*  1 Yes  2 No  *Have you been unfairly treated in the last 12 months*  *because of any other health problem or disability?*  1 Yes  2 No  *Have you been unfairly treated in the last 12 months*  *because of your sexual orientation?*  1 Yes  2 No | Binary:  None vs. Any form of discrimination |  |
| **Bullying** | SHOW CARD N5  Now looking at this card, could you tell me if you have ever experienced any of these problems or events, at any time in your life:  **1 Bullying**  2 Violence at work  3 Violence in the home  4 Sexual abuse  5 Being expelled from school  6 Running away from your home  7 Being homeless  8 None of these | Binary:  No v yes |  |
| ***Health behaviours*** |  |  |  |
| **Current smoker** | Have you ever smoked a cigarette?  *INTERVIEWER: Please do not include electronic cigarettes*  1 Yes  2 No  IF Yes  Do you smoke cigarettes at all nowadays?  1 Yes  2 No | Binary:  No vs yes | Derived variable based on responses to |
| **Problem drinking** | *1) In the last 12 months, how often have you had a drink containing alcohol?*  1 Never  2 Monthly  3 Two to four times a month  4 Two to three times a week  5 Four or more times a week  *2) How many standard drinks containing alcohol do you have on a typical day when you are drinking?*  A standard drink is half a pint of beer, a single measure of spirits or a small glass of wine.  1 One or two  2 Three or four  3 Five or six  4 Seven, eight, or nine  5 Ten or more  *3) Thinking about your drinking in the last year, how often do you have 6 or more drinks on one occasion?*  *4) How often during the last year have you found that you were not able to stop drinking once you had started?*  *5) How often during the last year have you failed to do what was normally expected from you because of drinking?*  *6) How often during the last year have you needed a first drink in the morning to get yourself going after a heavy drinking session?*  *7) How often during the last year have you had a feeling of guilt or remorse after drinking?*  *8) How often during the last year have you been unable to remember what happened the night before because*  *you had been drinking?*  **For each of the Qs 3 to 8 above, options are:**  **1 Never**  **2 Less than monthly,**  **3 Monthly,**  **4 Weekly**  **5 Daily or almost daily**  *9) Have you or someone else been injured as a result of your drinking?*  1 Yes, but not in the last year  2 Yes, during the last year  3 No  *10) Has a relative, a friend, or a doctor or other health worker been concerned about your drinking or suggested*  *you cut down?*  1 Yes, but not in the last year  2 Yes, during the last year  3 No | Binary:  No (AUDIT score<8, low risk) vs yes (AUDIT score ≥8, increasing risk) | Derived variable (based on AUDIT scores) already available in the dataset  Based on answers to the 10 questions in the AUDIT screening tool, total scores are calculated (ranging from minimum 0 to maximum 40). |
| **Drug frequency** | *How many times have you ever used* ***XX*** *drug*  1 less than 10 times  2 10 to 100 times  3 more than 100 times?  The same question was asked for each of the following types of recreational drugs:  Cannabis, Amphetamine, Cocaine, Crack, Ecstasy, Heroin/methadone/physeptone, Tranquillisers, and Glue/solvents/gas/aerosols | Binary:  No (<10 times) vs yes (≥10 times) | Single binary variable indicating <10 times vs ≥10 times of any drug use |
| **Drug dependency** | *In the past 12 months have you used* ***XX*** *drug to the extent that you felt like you needed it or were dependent on it?*  1 Dependent on cannabis only  2 Dependent on any OTHER drug  3 No dependency | Binary:  No dependency vs yes, dependency on any drug | Derived variable already available in dataset |

**Supplemental Table 2. Differences in prevalence of past experiences of homelessness by sexual identity in 10,428 individuals who took part in the 2007 and 2014 Adult Psychiatric Morbidity Survey (APMS)**

|  | **Model 1** | | **Model 2** | |
| --- | --- | --- | --- | --- |
|  | **PR** | **95% CI** | **PR** | **95% CI** |
| **Sexual identity** |  |  |  |  |
| Heterosexual | **1** |  | 1 |  |
| Bisexual | **2.58** | **1.49,4.48** | **2.52** | **1.48,4.29** |
| Gay/lesbian | **1.98** | **1.10,3.57** | 1.76 | 0.97,3.19 |
| Other | 1.24 | 0.64,2.37 | 1.10 | 0.57,2.13 |
| **Age (years)** |  |  |  |  |
| 16 - 24 |  |  | **0.65** | **0.45,0.93** |
| 25 - 34 |  |  | 1.22 | 0.95,1.57 |
| 35 - 44 |  |  | 1.12 | 0.87,1.44 |
| 45 - 64 |  |  | 1 |  |
| **Sex** |  |  |  |  |
| Female |  |  | 0.91 | 0.75-1.10 |
| **IMD** |  |  |  |  |
| Quintile 1 |  |  | 1 |  |
| Quintile 2 |  |  | **1.74** | **1.10,2.74** |
| Quintile 3 |  |  | **2.63** | **1.71,4.04** |
| Quintile 4 |  |  | **3.25** | **2.13,4.95** |
| Quintile 5 *(most disadvantaged)* |  |  | **4.38** | **2.91,6.58** |
| **Survey year** |  |  |  |  |
| 2007 |  |  | 1 |  |
| 2014 |  |  | 1.01 | 0.83-1.23 |

Model 1: Unadjusted, Model 2: Adjusted for age, sex, IMD and survey year. Text in bold text indicates 95%

confidence intervals that do not include 0.

**Supplemental Table 3. Differences in mental health outcomes by sexual identity and past experiences of homelessness in 10,428 individuals who took part in the 2007 and 2014 Adult Psychiatric Morbidity Survey (APMS)**

|  | **CMD** | | | | | | **GAD** | | | | | |
| --- | --- | --- | --- | --- | --- | --- | --- | --- | --- | --- | --- | --- |
|  | **Model 1** | | **Model 2** | | **Model 3** | | **Model 1** | | **Model 2** | | **Model 3** | |
| **Sexual identity** | **PR** | **95% CI** | **PR** | **95% CI** | **PR** | **95% CI** | **PR** | **95% CI** | **PR** | **95% CI** | **PR** | **95% CI** |
| **Hetero-NH** | 1 |  | 1 |  | 1 |  | 1 |  | 1 |  | 1 |  |
| **Hetero-H** | **2.68** | **2.38,3.03** | **2.49** | **2.20,2.81** | **1.82** | **1.61,2.05** | **3.54** | **2.77,4.52** | **3.34** | **2.59,4.30** | **2.27** | **1.74,2.96** |
| **SM-NH** | **1.82** | **1.45,2.28** | **1.76** | **1.40,2.20** | **1.27** | **1.02,1.59** | **1.87** | **1.19,2.95** | **1.79** | **1.13,2.82** | 1.21 | 0.77,1.91 |
| **SM-H** | **4.12** | **3.01,5.64** | **3.82** | **2.88,5.07** | **2.42** | **1.86,3.15** | **5.29** | **2.51,11.13** | **4.87** | **2.34,10.12** | **2.78** | **1.28,6.05** |
| **Other-NH** | 1.35 | 0.93,1.98 | 1.26 | 0.86,1.85 | 1.12 | 0.79,1.59 | 1.66 | 0.81,3.42 | 1.63 | 0.79,3.36 | 1.41 | 0.73,2.71 |
| **Other-H** | 4.01 | 2.53,6.38 | 3.3 | 1.93,5.63 | 2.64 | 1.17,5.95 | 3.21 | 0.56,18.53 | 2.98 | 0.54,16.45 | 2.2 | 0.31,15.63 |
| **Observations** | 10403 |  | 10403 |  | 10403 |  | 10403 |  | 10403 |  | 10403 |  |
|  | **Depression** | | | | | | **Traumatic experiences** | | | | | |
| **Sexual identity** | **PR** | **95% CI** | **PR** | **95% CI** | **PR** | **95% CI** | **PR** | **95% CI** | **PR** | **95% CI** | **PR** | **95% CI** |
| **Hetero-NH** | 1 |  | 1 |  | 1 |  | 1 |  | 1 |  | 1 |  |
| **Hetero-H** | **4.91** | **3.74,6.46** | **4.25** | **3.18,5.68** | **2.89** | **2.15,3.88** | **1.80** | **1.66,1.96** | **1.76** | **1.62,1.92** | **1.52** | **1.39,1.66** |
| **SM-NH** | **1.89** | **1.12,3.20** | **1.83** | **1.07,3.13** | 1.25 | 0.73,2.15 | **1.41** | **1.22,1.64** | **1.48** | **1.27,1.72** | **1.22** | **1.05,1.42** |
| **SM-H** | **8.85** | **4.40,17.78** | **7.85** | **3.98,15.48** | **4.66** | **2.28,9.51** | **2.38** | **1.99,2.84** | **2.43** | **2.02,2.92** | **1.88** | **1.53,2.30** |
| **Other-NH** | 1.75 | 0.83,3.71 | 1.56 | 0.75,3.26 | 1.30 | 0.60,2.84 | 0.71 | 0.51,0.99 | 0.68 | 0.49,0.95 | 0.66 | 0.48,0.92 |
| **Other-H** | **8.31** | **2.36,29.21** | **6.89** | **2.09,22.68** | **5.07** | **1.16,22.20** | 1.63 | 0.94,2.85 | 1.55 | 0.88,2.71 | 1.57 | 0.91,2.70 |
| **Observations** | 10403 |  | 10403 |  | 10403 |  | 10181 |  | 10181 |  | 10181 |  |
|  | **Phobia** | | | | |  | **Self-harm, lifetime** | | | | | |
| **Sexual identity** | **PR** | **95% CI** | **PR** | **95% CI** | **PR** | **95% CI** | **PR** | **95% CI** | **PR** | **95% CI** | **PR** | **95% CI** |
| **Hetero-NH** | 1 |  | 1 |  | 1 |  | 1 |  | 1 |  | 1 |  |
| **Hetero-H** | **5.78** | **4.28,7.81** | **5.10** | **3.80,6.87** | **3.36** | **2.43,4.65** | **4.90** | **4.00,6.01** | **4.82** | **3.95,5.87** | **3.20** | **2.58,3.97** |
| **SM-NH** | **2.58** | **1.46,4.55** | **2.35** | **1.35,4.08** | 1.47 | 0.83,2.60 | **4.13** | **3.11,5.50** | **3.20** | **2.46,4.17** | **2.09** | **1.60,2.73** |
| **SM-H** | **7.59** | **2.85,20.17** | **6.51** | **2.82,15.03** | **3.54** | **1.32,9.51** | **7.82** | **4.55,13.41** | **6.92** | **4.44,10.79** | **3.82** | **2.60,5.60** |
| **Other-NH** | 1.24 | 0.42,3.65 | 1.10 | 0.37,3.26 | 0.88 | 0.29,2.68 | 1.86 | 0.98,3.50 | **1.91** | **1.03,3.55** | 1.63 | 0.95,2.79 |
| **Other-H** | 3.64 | 0.54,24.76 | 2.66 | 0.36,19.68 | 1.86 | 0.22,15.69 | 3.66 | 0.96,13.86 | **2.97** | **1.07,8.26** | 1.88 | 0.92,3.82 |
| **Observations** | 10403 |  | 10403 |  | 10403 |  | 10401 |  | 10401 |  | 10401 |  |
|  | **Attempted suicide, lifetime** | | | | | |  |  |  |  |  |  |
| **Sexual identity** | **PR** | **95% CI** | **PR** | **95% CI** | **PR** | **95% CI** |  |  |  |  |  |  |
| **Hetero-NH** | 1 |  | 1 |  | 1 |  |  |  |  |  |  |  |
| **Hetero-H** | **3.18** | **2.84,3.55** | **3.07** | **2.74,3.44** | **2.24** | **1. 99,2.53** |  |  |  |  |  |  |
| **SM-NH** | **2.37** | **1.98,2.84** | **2.20** | **1.85,2.61** | **1.62** | **1.35,1.94** |  |  |  |  |  |  |
| **SM-H** | **5.05** | **4.17,6.11** | **4.73** | **3.93,5.70** | **3.03** | **2.38,3.84** |  |  |  |  |  |  |
| **Other-NH** | 1.16 | 0.81,1.68 | 1.17 | 0.81,1.70 | 1.07 | 0.74,1.55 |  |  |  |  |  |  |
| **Other-H** | 2.07 | 0.90,4.77 | 2.0 | 0.96,4.19 | 1.57 | 0.83,2.96 |  |  |  |  |  |  |
| **Observations** | 10395 |  | 10395 |  | 10395 |  |  |  |  |  |  |  |

Model 1: Unadjusted Poisson regression. Model 2: Poisson regression adjusted for age, sex, socioeconomic deprivation (IMD) and sweep (2007 or 2014). Model 3: Poisson regression adjusted for model 2 + adjustment for discrimination and bullying. Note: NH: no experiences of past homelessness, H: past experiences of homelessness, SM: sexual minority. Text in bold text indicates 95% confidence intervals that do not include 0. CMD: Common mental disorders, GAD: Generalised anxiety disorder.

**Supplemental Table 4.** **Differences in health behaviours by sexual identity and past experiences of homelessness in 10,428 individuals who took part in the 2007 and 2014 Adult Psychiatric Morbidity Survey (APMS)**

|  | **Current smoker** | | | | | | **Drinking problem** | | | | | |
| --- | --- | --- | --- | --- | --- | --- | --- | --- | --- | --- | --- | --- |
|  | **M1** |  | **M2** |  | **M3** |  | **M1** |  | **M2** |  | **M3** |  |
| **Sexual identity** | **PR** | **95% CI** | **PR** | **95% CI** | **PR** | **95% CI** | **PR** | **95% CI** | **PR** | **95% CI** | **PR** | **95% CI** |
| **Hetero-NH** | 1 |  | 1 |  | 1 |  | 1 |  | 1 |  | 1 |  |
| **Hetero-H** | **2.21** | **1.98,2.46** | **1.95** | **1.75,2.17** | **1.92** | **1.72,2.14** | **1.23** | **1.03,1.45** | **1.24** | **1.05,1.45** | **1.20** | **1.02,1.42** |
| **SM-NH** | **1.50** | **1.23,1.85** | **1.42** | **1.16,1.73** | **1.40** | **1.14,1.71** | **1.45** | **1.16,1.81** | **1.41** | **1.14,1.74** | **1.37** | **1.11,1.70** |
| **SM-H** | **2.72** | **1.91,3.87** | **2.50** | **1.70,3.67** | **2.45** | **1.66,3.59** | 1.64 | 0.96,2.79 | **1.71** | **1.02,2.88** | 1.64 | 0.97,2.80 |
| **Other-NH** | 0.91 | 0.63,1.34 | 0.83 | 0.56,1.22 | 0.83 | 0.56,1.23 | 0.74 | 0.47,1.15 | 0.74 | 0.48,1.14 | 0.73 | 0.48,1.13 |
| **Other-H** | 2.13 | 1.09,4.19 | 1.73 | 0.91,3.29 | 1.72 | 0.90,3.26 | 1.24 | 0.44,3.46 | 1.25 | 0.58,2.69 | 1.23 | 0.56,2.70 |
| **Observations** | 10403 |  | 10403 |  | 10403 |  | 10391 |  | 10391 |  | 10391 |  |
|  | **Drug dependence** | | | | | | **Drug frequency** | | | | | |
|  | **M1** |  | **M2** |  | **M3** |  | **M1** |  | **M2** |  | **M3** |  |
| **Sexual identity** | **PR** | **95% CI** | **PR** | **95% CI** | **PR** | **95% CI** | **PR** | **95% CI** | **PR** | **95% CI** | **PR** | **95% CI** |
| **Hetero-NH** | 1 |  | 1 |  | 1 |  | 1 |  | 1 |  | 1 |  |
| **Hetero-H** | **5.15** | **3.84,6.90** | **4.78** | **3.64,6.26** | **4.03** | **3.00,5.42** | **3.32** | **2.74,4.01** | **3.19** | **2.65,3.83** | **2.85** | **2.36,3.44** |
| **SM-NH** | **3.08** | **1.83,5.19** | **2.73** | **1.64,4.52** | **2.19** | **1.27,3.79** | **2.98** | **2.27,3.91** | **2.62** | **2.00,3.43** | **2.29** | **1.72,3.05** |
| **SM-H** | **8.53** | **3.99,18.24** | **9.81** | **4.44,21.67** | **7.38** | **3.15,17.29** | **4.44** | **2.54,7.73** | **4.96** | **2.77,8.86** | **4.06** | **2.19,7.55** |
| **Other-NH** | 1.61 | 0.71,3.67 | 1.33 | 0.59,3.01 | 1.18 | 0.52,2.66 | 0.59 | 0.27,1.31 | 0.53 | 0.24,1.15 | 0.49 | 0.22,1.06 |
| **Other-H** | 2.42 | 0.35,16.59 | 1.55 | 0.21,11.38 | 1.48 | 0.21,10.56 | 2.71 | 0.77,9.46 | 1.87 | 0.74,4.73 | 1.78 | 0.63,5.01 |
| **Observations** | 10256 |  | 10256 |  | 10256 |  | 10399 |  | 10399 |  | 10399 |  |

Model 1: Unadjusted Poisson regression. Model 2: Poisson regression adjusted for age, sex, socioeconomic deprivation (IMD) and sweep (2007 or 2014). Model 3: Poisson regression adjusted for model 2 + adjustment for discrimination and bullying. Note: NH: no experiences of past homelessness, H: past experiences of homelessness, SM: sexual minority. Text in bold text indicates 95% confidence intervals that do not include 0.

**Supplemental Figure 1. Prevalence of mental health problems based on past experiences of homelessness and sexual identity in 10,428 individuals who answered the 2007 or 2014 Adult Psychiatric Morbidity Surveys. Estimates based on Model 3 in Supplemental Table 3**

**
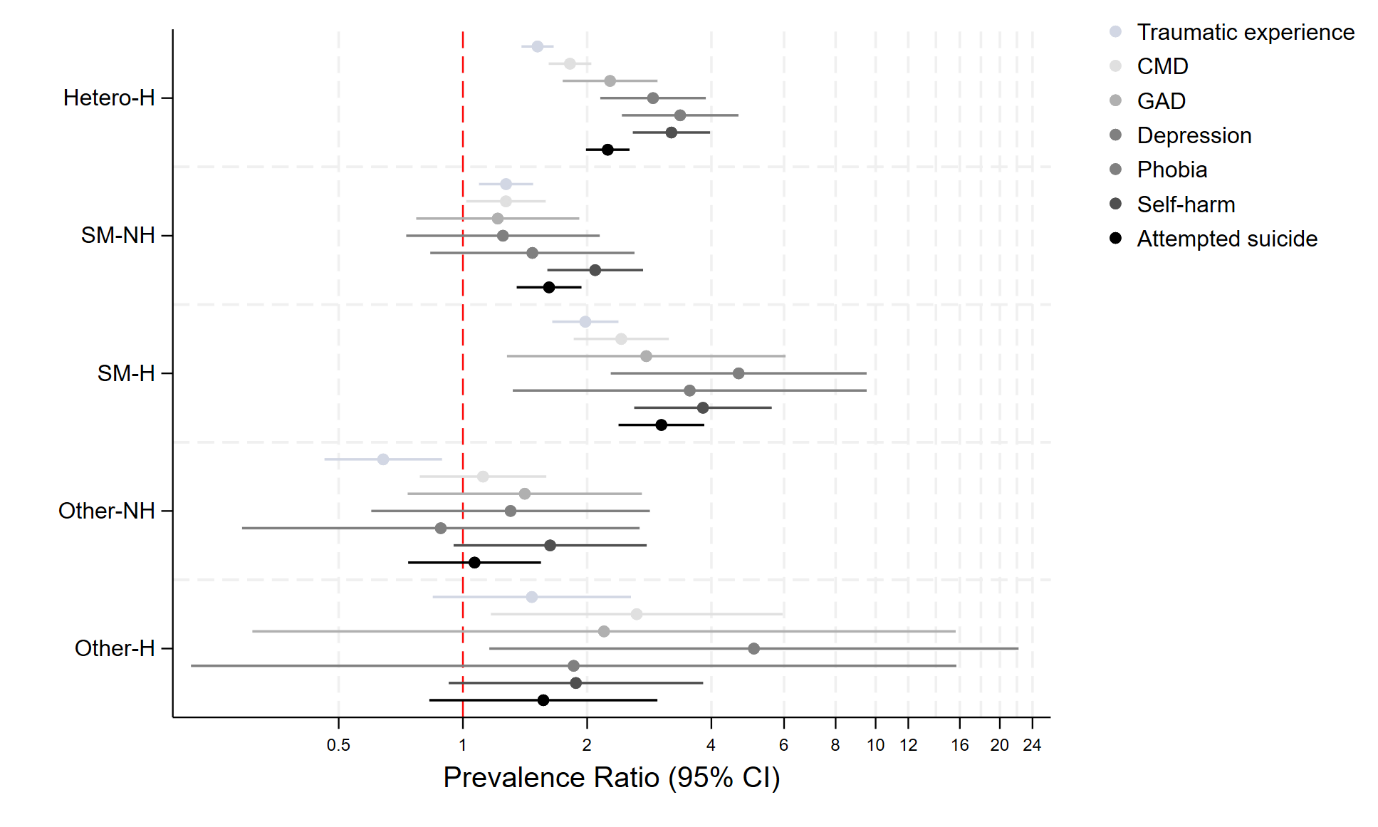
**

Legend:

Hetero-H: Heterosexual and past homelessness

SM-NH: Sexual minority and no past homelessness

SM-H: Sexual minority and past homelessness

Other-NH: Other sexual identity and no past homelessness

Other-H: Other sexual identity and past homelessness

Reference category: Heterosexual individuals and no past homelessness
